# Socioeconomic determinants of excess weight and obesity among Indigenous women: findings from the First National Survey of Indigenous People’s Health and Nutrition in Brazil

**DOI:** 10.1017/S1368980020000610

**Published:** 2021-05

**Authors:** Carlos EA Coimbra, Felipe G Tavares, Aline A Ferreira, James R Welch, Bernardo L Horta, Andrey M Cardoso, Ricardo Ventura Santos

**Affiliations:** 1 Escola Nacional de Saúde Pública, Fundação Oswaldo Cruz, Rio de Janeiro, RJ 21041-210, Brazil; 2 Escola de Enfermagem Aurora de Afonso Costa, Universidade Federal Fluminense, Niterói, RJ 24020-091, Brazil; 3 Instituto de Nutrição Josué de Castro, Universidade Federal do Rio de Janeiro, Rio de Janeiro, RJ 21941-902, Brazil; 4 Programa de Pós-Graduação em Epidemiologia, Universidade Federal de Pelotas, Pelotas, RS 96001-970, Brazil; 5 Departamento de Antropologia, Museu Nacional, Universidade Federal do Rio de Janeiro, Rio de Janeiro, RJ 20940-040, Brazil

**Keywords:** Nutrition assessment, Obesity, Women, Socioeconomic factors, Indigenous peoples

## Abstract

**Objective:**

This article assesses the nutritional status of Indigenous women from 14 to 49 years of age in Brazil.

**Design:**

Sample size was calculated for each region considering a prevalence of 50 % for all disease outcomes, a relative error of 5 % and a CI of 95 %. In the initial data analysis, the prevalence of excess weight and obesity was calculated according to independent variables. Multivariate multilevel hierarchical analyses were conducted based on a theoretical model of two ranked blocks.

**Setting:**

The 2010 Indigenous population in Brazil was 896 000, with approximately 300 Indigenous ethnic groups, making Brazil one of the most ethnically diverse countries in the Americas and the world.

**Participants:**

Of the total target sample of 6722 women evaluated by the National Survey, thirty did not participate, 939 were not eligible for analyses due to pregnancy or unknown pregnancy status, and thirty-nine were excluded due to missing anthropometric data.

**Results:**

The evaluation of nutritional status was completed for 5714 non-pregnant women (99·3 % of eligible participants for this outcome). High prevalence rates were encountered for both excess weight (46·2 %) and obesity (15·8 %) among the sampled women. In the multivariate analyses, higher socioeconomic indicators, market-integrated living conditions and less reliance on local food production, as well as increased age and parity were associated with excess weight and obesity.

**Conclusion:**

Results point to distinct patterns of associations between socioeconomic indicators and the occurrence of excess weight and obesity among Indigenous women, which have potentially significant implications from a public policy perspective for Indigenous peoples in Brazil.

Globally, questions about excess weight in human populations are among the most urgent of contemporary public health priorities. Accelerated increases in overweight and obesity prevalence during recent decades are demonstrated for diverse countries worldwide^(1–3)^. Overweight and obesity are implicated as risk factors for a set of linked chronic non-communicable diseases, such as CVD, diabetes mellitus and some kinds of cancer^(4,5)^.

According to Stevens, in 2008 approximately one in nine adults worldwide was obese, and at least one in five women in 117 countries was obese^(3)^. In some low- and medium-income countries, the nutritional transition has progressed at an even more accelerated rate than has been observed globally due to recent socioeconomic and environmental changes that contribute to rural–urban migration and conversion, substitution of traditional diets, and increased physical inactivity, among other factors associated with excess weight^(6,7)^. In Brazil, nutrition transition has been observed since the mid-1970s along with highly unequal distributions of obesity and chronic diseases following income, education, race or ethnicity and other sociodemographic indicators^(8,9)^. Recent surveys of physical activity in Brazil showed relatively low proportions of male and female adults considered to be physically active^(10–12)^.

Until recently, few studies compared the health of Indigenous populations internationally^(13)^, although numerous investigations showed that exposure to social and environmental factors associated with nutrition transition, especially obesity, is a common occurrence. For example, Story reported that in North America ‘obesity has become a major health problem in American Indians only in the past 1–2 generations and is believed to be associated with the relative abundance of high-fat foods and the rapid change from active to sedentary lifestyles’^(14)^ (p. 747S). In other countries and regions with large Indigenous populations, such as Canada, Australia, the Pacific Islands and Greenland, obesity and associated metabolic and cardiovascular diseases also stand out as important public health issues^(15–18)^. Although epidemiological information for Latin America is more dispersed, recent analyses indicated that obesity among Indigenous peoples is increasing rapidly in association with cardiovascular and metabolic diseases^(19–21)^.

The appearance and rapid increase in obesity, diabetes mellitus and arterial hypertension prevalence among Indigenous peoples in Brazil has been observed since the 1970s and 1980s, especially in the Amazon region^(22–24)^. More recently, case studies have documented elevated prevalence of excess weight in diverse Indigenous ethnic groups located throughout the country^(25–30)^. At the same time, this population continues to suffer from a high prevalence of chronic undernutrition and of preventable infectious and parasitic diseases affecting mostly children^(31–33)^.

The epidemiology of excess weight and obesity in Brazil is the focus of growing attention, but no previous study has addressed the subject for Indigenous peoples nationally. The First National Survey of Indigenous People’s Health and Nutrition in Brazil (henceforth ‘National Survey’), conducted in 2008–9, was the first study to evaluate a nationwide representative sample of Indigenous peoples throughout the country. It is the most comprehensive investigation yet conducted on the nutritional situation of Indigenous adult women in Brazil, and one of the most extensive and detailed study ever carried out in Latin America^(34)^. The present article assesses the nutritional status of Indigenous women from 14 to 49 years of age in Brazil.

## Methods

A stratified probability sample of the country’s official geopolitical regions (North, Northeast, Central-West and South/Southeast; the South and Southeast regions were joined due to their substantially smaller Indigenous populations) was obtained using multistage sampling based on a list of Indigenous villages provided in 2008 by the Brazilian National Health Foundation^(34)^. Of the list’s 3995 villages located in federally recognised Indigenous Reserves, 1227 were excluded due to indications that they were vacated, deactivated or otherwise unstable (151 villages), or had <31 inhabitants (1076 villages), which was the minimum village size investigated.

Sample size was calculated for each region considering a prevalence of 50 % for all disease outcomes, a relative error of 5 % and a CI of 95 %^(35)^. Women aged 14–49 years were considered adults and included in the analyses (see Coimbra *et al*.^(34)^ for a detailed sampling procedure). Interviews and clinical examinations were conducted in women’s homes, in Portuguese, with local Indigenous translators when needed.

Body weight was measured to the nearest 100 g with a portable digital scale (Seca model 872) and with participants wearing minimal clothing and without footwear. Standing height was measured with an AlturaExata portable anthropometer and recorded to the nearest 0·1 cm. Anthropometric measurements were carried out by trained and standardised field researchers^(34)^.

In accordance with WHO guidelines, BMI of women ≥20 years of age was classified as underweight (<18·5 kg/m^2^), normal weight (18·5–24·9 kg/m^2^), overweight (25·0–29·9 kg/m^2^) and obese (≥30 kg/m^2^)^(36)^. For participants aged 14–19 years, cut-off points proposed by de Onis^(37)^ were followed. Excess weight was calculated as the sum of overweight and obesity frequencies. Data for pregnant women and women with unreported pregnancy status were excluded from the analysis.

In the initial data analysis, the prevalence of excess weight and obesity was calculated according to independent variables (village, household and women’s individual demographic, socioeconomic and reproductive characteristics).

Contextual village-level independent variables included geopolitical region, village food acquisition activities (hunting, fishing and collecting) and presence of a school meals programme.

Household-level variables included regular income (presence of income from salaries, pensions or social benefits), several composite socioeconomic indicators derived through principal component analyses (see below), number of household residents, access to indoor electricity, household food acquisition activities (horticulture and livestock, and purchasing), access to a staple food donation programme (*cesta básica*), a purchased foods index derived through principal component analyses (see below), type of cooking fuel and use of oils and fats for cooking.

A principal component analysis was used to create the following socioeconomic and living condition variables: (a) household goods index, based on the quantities of durable industrialised goods in each household (first component explained 19 % of variance, eigenvalue 3·56); (b) housing conditions index, based on the type of flooring, walls, roofing, presence of electricity and fuel used for cooking (first component explained 48·0 % of variance, eigenvalue 1·44); and (c) sanitation index, based on the primary defecation location, trash disposal destination, primary source of drinking water and availability of filtered water for drinking in the house (first component explained 56·5 % of variance, eigenvalue 0·63). Households received scores based on the sum of the contribution of each item multiplied by the quantity of each item before being classified according to terciles of the overall distribution, considering the four regions combined. In each case, the first tercile indicates a lower index score associated with fewer durable industrialised goods, less access to ‘high-quality’ housing materials and resources and lower-quality sanitation conditions.

A principal component analysis was also utilised to derive a household purchased foods acquisition index using fourteen food items: rice, corn, manioc, tubers (sweet potatoes and yams), beans, fruits, nuts, vegetables, milk, egg, chicken, beef, game and fish. Varimax rotation was used to improve component interpretation. Food items were considered representative of each component if they showed a loading >0·3. The first component, which explained 23·3 % of variance (eigenvalue 3·26), was dominated by predominantly purchased food items (rice, beans, milk, egg, chicken and beef), whereas the second and third components (not utilised in the analyses) were dominated by locally produced and acquired foods. As above, household purchased foods acquisition index scores were the sum of the contribution of each item multiplied by item quantity, classified according to terciles of the combined distribution.

Women’s individual variables included age group, years of schooling and number of children ever born (parity).

Multivariate multilevel hierarchical analyses were conducted based on a theoretical model of two ranked blocks comprising (1) village and (2) household and individual characteristics. All estimates were made using *xtme* family commands and were corrected for the complex sampling design using Stata survey commands (*svy*)^(38)^. The association of each independent variable with the presence of excess weight and obesity was estimated using logistic regression, adjusting for region of residence. Independent variables with *P*-values <0·20 in this analysis were selected for further multivariate hierarchical analyses. Variables with at least one category with *P* ≤ 0·20 for excess weight or obesity are indicated in Tables 1–3.

**Table 1:** Prevalence and OR (from logistic regression) for excess weight and obesity of Indigenous women 14–49 years of age, according to region and village characteristics (First National Survey of Indigenous People’s Health and Nutrition in Brazil, 2008–9)

Variable	Excess weight*				Obesity†			
	*n*	Prevalence (%)	OR	95 % CI	*n*	Prevalence (%)	OR	95 % CI
Region								
North	645	31·2	1·0	reference	125	6·1	1·0	reference
Central-West	591	52·8	2·5	1·66, 3·68	192	17·2	3·2	1·58, 6·37
Northeast	651	41·3	1·6	1·04, 2·32	213	13·5	2·4	1·21, 4·74
South/Southeast	517	54·7	2·7	1·49, 4·78	212	22·6	4·5	1·95, 10·17
Brazil	2404	46·2			742	15·8		
Hunting								
Yes	1728	45·8	1·0	reference	519	15·3	1·0	reference
No	676	47·3	1·1	0·68, 1·63	223	16·8	1·1	0·57, 2·17
Fishing								
Yes	2178	45·6	1·0	reference	648	15·3	1·0	reference
No	226	53·5	1·4	0·96, 1·96	94	21·4	1·5	0·95, 2·40
Food collection								
Yes	1938	45·0	1·0	reference	564	15	1·0	reference
No	466	51·1	1·3	0·95, 1·82	178	19·3	1·4	0·92, 2·01
School meals programme								
Yes	2047	47·2	1·0	reference	652	16·5	1·0	reference
No	357	40·5	0·8	0·57, 1·02	90	11·3	0·6	0·43, 0·95

* BMI ≥ 25 kg/m^2^.

† BMI ≥ 30 kg/m^2^.

**Table 2:** Prevalence and OR (from logistic regression) for excess weight and obesity of Indigenous women 14–49 years of age, according to household characteristics (First National Survey of Indigenous People’s Health and Nutrition in Brazil, 2008–9)

Variable	Excess weight*				Obesity†			
	*n*	Prevalence (%)	OR	95 % CI	*n*	Prevalence (%)	OR	95 % CI
Regular income								
Yes	2087	47·2	1·3	1·02, 1·67	655	16·2	1·3	0·97, 1·79
No	316	40·5	1·0	reference	87	12·9	1·0	reference
Household goods index				*P* ≤ 0·20				*P* ≤ 0·20
1º tercile (low)	602	37·4	1·0	reference	130	9·9	1·0	reference
2º tercile	803	46·3	1·4	1·22, 1·71	246	16·4	1·8	1·37, 2·33
3º tercile (high)	999	52·5	1·9	1·52, 2·25	366	19·5	2·2	1·54, 3·20
Housing conditions index				*P* ≤ 0·20				*P* ≤ 0·20
1º tercile (low)	684	40·3	1·0	reference	152	9·7	1·0	reference
2º tercile	773	48·7	1·4	0·99, 2·00	255	19·5	2·3	1·42, 3·59
3º tercile (high)	938	49·0	1·4	1·13, 1·79	332	17·6	2·0	1·55, 2·57
Sanitation index				*P* ≤ 0·20				*P* ≤ 0·20
1º tercile (low)	938	42·7	1·0	reference	256	14·1	1·0	reference
2º tercile	657	46·7	1·2	0·86, 1·60	201	15·3	1·1	0·70, 1·75
3º tercile (high)	794	50·0	1·3	0·99, 1·80	282	18·3	1·4	0·88, 2·12
Number of household residents				*P* ≤ 0·20				*P* ≤ 0·20
1–3	337	44·6	1·0	reference	101	13·4	1·0	reference
4–6	1036	48·9	1·2	1·00, 1·42	351	17·6	1·4	1·06, 1·78
7–9	706	47·2	1·1	0·90, 1·37	199	15·3	1·2	0·89, 1·53
≥10	318	37·9	0·8	0·55, 1·04	88	13·6	1·0	0·62, 1·65
Access to indoor electricity				*P* ≤ 0·20				*P* ≤ 0·20
Yes, continuous	1711	49·2	1·7	1·30, 2·21	601	18·4	2·7	1·85, 4·04
Yes, discontinuous	294	41·2	1·2	0·74, 2·03	67	10·2	1·4	0·70, 2·75
No	397	36·4	1·0	reference	74	7·6	1·0	reference
Horticulture and livestock								
Yes	2019	45·9	1·0	reference	623	15·8	1·0	reference
No	384	48·2	1·1	0·91, 1·32	119	16·0	1·0	0·82, 1·26
Hunting and fishing				*P* ≤ 0·20				
Yes	1425	42·5	1·0	reference	399	13·6	1·0	reference
No	978	51·3	1·4	1·12, 1·81	343	18·8	1·5	1·02, 2·13
Food collection				*P* ≤ 0·20				*P* ≤ 0·20
Yes	1603	43·4	1·0	reference	460	14·0	1·0	reference
No	800	52·0	1·4	1·15, 1·74	282	19·5	1·5	1·10, 2·01
Food purchasing				*P* ≤ 0·20				*P* ≤ 0·20
Yes	2333	46·5	1·4	0·84, 2·27	737	16·2	7·7	1·93, 30·66
No	70	38·5	1·0	reference	5	2·4	1·0	reference
Access to staple foods donation programme								
Yes	1116	50·8	1·5	1·15, 1·92	381	18·2	1·5	1·05, 2·07
No	1287	41·0	1·0	reference	361	13·1	1·0	reference
Purchased foods acquisition index								
1º tercile (low)	754	44·3	1·0	reference	213	14·9	1·0	reference
2º tercile	920	44·2	1·0	0·81, 1·23	269	14·1	0·9	0·75, 1·17
3º tercile (high)	727	50·3	1·3	1·03, 1·58	260	18·6	1·3	1·03, 1·64
Type of cooking fuel				*P* ≤ 0·20				*P* ≤ 0·20
Propane	727	51·8	1·4	1·12, 1·67	254	19·0	1·4	1·10, 1·73
Others (firewood, etc.)	1676	44·0	1·0	reference	488	14·5	1·0	reference
Use of oils and fats for cooking								
Yes	808	51·1	1·4	0·97, 1·94	282	20·0	1·6	1·02, 2·57
No	1510	43·2	1·0	reference	444	13·4	1·0	reference

* BMI ≥ 25 kg/m^2^

† BMI ≥ 30 kg/m^2^

**Table 3:** Prevalence and OR (from logistic regression) for excess weight and obesity of Indigenous women 14–49 years of age, according to individual characteristics (First National Survey of Indigenous People’s Health and Nutrition in Brazil, 2008–9)

Variable	Excess weight*				Obesity†			
	*n*	Prevalence (%)	OR	95 % CI	*n*	Prevalence (%)	OR	95 % CI
Age (years)				*P* ≤ 0·20				*P* ≤ 0·20
14–19	355	28·5	1·0	reference	49	4·1	1·0	reference
20–24	395	37·0	1·5	1·25, 1·73	105	11·6	3·1	2·13, 4·42
25–29	469	52·8	2·8	2·25, 3·49	148	17·9	5·1	3·44, 7·54
30–34	435	59·4	3·7	2·84, 4·74	153	24·1	7·4	5·37, 10·23
35–39	329	59·9	3·7	2·84, 4·95	130	25·6	8·1	5·34, 12·17
40–44	241	61·7	4·0	3·00, 5·41	82	22·8	6·9	4·94, 9·63
45–49	180	60·9	3·9	3·06, 4·99	75	29·0	9·6	6·36, 14·44
Years of schooling				*P* ≤ 0·20				*P* ≤ 0·20
0	373	51·7	1·7	1·21, 2·42	118	19·4	1·8	1·02, 3·01
1–4	995	50·0	1·6	1·26, 2·03	337	18·4	1·7	1·20, 2·26
5–9	623	43·5	1·2	1·01, 1·50	165	12·9	1·1	0·79, 1·47
≥10	402	38·5	1·0	reference	119	12·1	1·0	reference
Parity				*P* ≤ 0·20				*P* ≤ 0·20
0	330	31·2	1·0	reference	56	5·8	1·0	reference
1–2	621	40·2	1·5	1·21, 1·82	186	12·6	2·3	1·60, 3·37
3–4	600	54·1	2·6	2·09, 3·23	211	20·8	4·3	2·91, 6·20
≥5	848	58·3	3·1	2·48, 3·80	2285	22·9	4·8	3·47, 6·59

* BMI ≥ 25 kg/m^2^.

† BMI ≥ 30 kg/m^2^.

Independent variables entered the multivariate analysis with their respective blocks in the theoretical hierarchical model, from distal block 1 to proximal block 2. For the first block, selected village variables (*P* < 0·20) were jointly included in the model, controlling for geopolitical region. A backward procedure was then used to progressively exclude variables, retaining only those with a significance level of *P* < 0·05. These procedures were the same for the second block, which included household and individual variables. Therefore, in the final model, the OR and respective 95 % CI were simultaneously adjusted for variables with *P* < 0·05 in the same block of variables, those retained in the previous block, and geopolitical region. We also computed OR with 90 % CI but found that results did not differ from those obtained using 95 % CI. All analyses were conducted with Stata 14.0^(38)^.

The National Committee on Research Ethics (Comissão Nacional de Ética em Pesquisa – CONEP) and the National Indian Foundation (Fundação Nacional do Índio – FUNAI) approved the study. In each Indigenous community, the field team held meetings with leaders and other community members to present and explain study objectives and procedures. A free and informed collective consent form was signed by leaders and other community representatives of villages that decided to participate. All villages, households or guardians could decline to participate at any time.

## Results

Of the total target sample of 6722 women aged between 14 and 49 years evaluated by the National Survey, twenty-one did not participate due to absence, three due to declination and six for other reasons. Of 6692 participating women, 653 were not eligible for the present analyses due to pregnancy and 286 due to unknown pregnancy status. Of the 5753 remaining eligible women, thirty-nine were excluded due to missing anthropometric data. The evaluation of nutritional status was completed for 5714 women (99·3 % of eligible participants for this outcome).

BMI varied from 14·4 to 55·2 kg/m^2^, with a median of 24·0 kg/m^2^ (IQR 21·6–27·3). Comparing the geopolitical regions addressed in the study, the highest median was encountered in the South/Southeast (25·3 kg/m^2^, IQR 22·0–29·3) and the lowest in the North, with a median of 23·1 kg/m^2^ (IQR 21·2–25·6).

The overall prevalence of excess weight and obesity was, respectively, 46·2 and 15·8 % (Table 1). The highest prevalence of excess weight was recorded in the South/Southeast (54·7 %), followed by the Central-West (52·8 %), Northeast (41·3 %) and North (31·2 %) regions. The prevalence of obesity was distributed by region in the same order as described for excess weight: South/Southeast (22·6 %), Central-West (17·2 %), Northeast (13·5 %) and North (6·1 %). The only contextual village-level variable associated with excess weight based on a bivariate analysis of OR was geopolitical region. The only village-level variables showing a statistically significant association with obesity based on OR and CI were region and presence of a school meals programme (Table 1).

Of household-level variables (Table 2), only the sanitation index and presence of horticulture and livestock food acquisition were not associated with excess weight or obesity based on OR. The number of household residents, food purchasing and use of oils and fats for cooking were only associated with obesity. The overall observed pattern is that higher socioeconomic indicators, market-integrated living conditions and more reliance on purchased foods were associated with excess weight and obesity.

Concerning women’s individual descriptive analyses (Table 3), the risk of excess weight and obesity tended to increase with age, although the pattern was more consistent for excess weight. Women with more years of schooling presented lower rates of excess weight and obesity. For both outcomes, OR increased with parity.

The following variables remained in the final multilevel model for excess weight (95 % CI): geopolitical region, household good index, age group and years of schooling (Table 4). Except for schooling, the same set of variables was retained in the multivariate model for obesity, along with household food purchasing, housing conditions index and parity (Table 5). In both models, the Central-West and South/Southeast regions presented the highest OR. Women living in the Central-West, Northeast and South/Southeast regions were nearly 4·30, 2·89 and 5·64 times more likely to be obese compared to those living in the North. The overall pattern observed in the multivariate analyses is that higher socioeconomic indicators, market-integrated living conditions and less reliance on local food production, as well as increased age and parity were associated with excess weight and obesity.

**Table 4:** OR and 95 % CI for variables retained in the multilevel statistical models for excess weight (BMI ≥ 25 kg/m^2^) (First National Survey of Indigenous People’s Health and Nutrition in Brazil, 2008–9)

Variable	Geopolitical region		Model 1*		Model 2†	
	OR	95 % CI	OR	95 % CI	OR	95 % CI
Geopolitical region						
North	1·00	reference	1·00	reference	1·00	reference
Central-West	2·75	1·71, 4·40	2·58	1·64, 4·06	3·16	1·85, 5·40
Northeast	1·50	1·02, 2·21	1·25	0·85, 1·81	1·32	0·85, 2·05
South/Southeast	2·95	1·93, 4·51	2·53	1·68, 3·80	3·09	1·90, 5·02
Household goods index						
1º tercile (low)			1·00	reference	1·00	reference
2º tercile			1·40	1·19, 1·66	1·48	1·22, 1·79
3º tercile (high)			1·72	1·43, 2·06	1·84	1·48, 2·28
Age group						
14–19					1·00	reference
20–24					1·69	1·36, 2·09
25–29					3·63	2·87, 4·60
30–34					5·11	3·94, 6·63
35–39					6·08	4·56, 8·12
40–44					6·72	4·85, 9·31
45–49					5·62	3·98, 7·96
Years of schooling						
0					1·00	reference
1–4					1·25	1·00, 1·56
5–9					1·24	0·96, 1·60
≥10					0·88	0·66, 1·18

* Model 1 includes village and household variables retained in the analysis, adjusted by region.

† Model 2 includes village, household and individual variables retained in the analysis, adjusted by region.

**Table 5:** OR and 95 % CI for variables retained in the multilevel statistical models for obesity (BMI ≥ 30 kg/m^2^) (First National Survey of Indigenous People’s Health and Nutrition in Brazil, 2008–9)

Variable	Geopolitical region		Model 1*		Model 2†	
	OR	95 % CI	OR	95 % CI	OR	95 % CI
Geopolitical region						
North	1·00	Reference	1·00	reference	1·00	reference
Central-West	4·30	2·25, 8·23	3·80	2·13, 7·45	4·96	2·32, 10·61
Northeast	2·89	1·66, 5·00	2·18	1·26, 3·79	2·29	1·19, 4·42
South/Southeast	5·64	3·13, 10·15	4·23	2·39, 7·51	5·32	2·65, 10·67
Household goods index						
1º tercile (low)			1·00	reference	1·00	reference
2º tercile			1·54	1·18, 2·01	1·70	1·24, 2·31
3º tercile (high)			1·98	1·49, 2·64	2·16	1·53, 3·03
Household food purchasing						
Yes			4·28	1·67, 10·98	4·91	1·74, 13·86
No			1·00	reference	1·00	reference
Housing conditions index						
1º tercile (low)			1·00	reference	1·00	Reference
2º tercile			1·35	1·01, 1·82	1·35	0·96, 1·92
3º tercile (high)			1·11	0·81, 1·51	1·12	0·78, 1·60
Age group						
14–19					1·00	reference
20–24					2·49	1·61, 3·86
25–29					5·10	3·16, 8·23
30–34					7·99	4·73, 13·52
35–39					10·72	6·09, 18·89
40–44					9·07	4·99, 16·49
45–49					11·91	6·38, 22·25
Parity						
0					1·00	reference
1–2					1·49	0·99, 2·23
3–4					1·72	1·10, 2·67
≥5					1·42	0·89, 2·27

* Model 1 includes village and household variables retained in the analysis, adjusted by region.

† Model 2 includes village, household and individual variables retained in the analysis, adjusted by region.

## Discussion

Recently published studies present important international and global comparative analyses of the health and nutrition of Indigenous peoples^(13,19,39,40)^. Nutrition transition is a principal focus of these investigations due to the growing relevance of obesity and comorbidities for the determination of illness and death in Indigenous populations. Nevertheless, it is important to recall that in diverse world regions such as Latin America, food insecurity, child undernutrition and elevated burden of infectious and parasitic diseases persist in Indigenous peoples’ epidemiological profiles, generally reaching much higher levels than those recorded for their respective benchmark national populations^(13,40–42)^.

Based on a global comparative analysis of Indigenous peoples’ health, obesity emerges as one of the most worrying health problems. It affects 50–70 % of the Indigenous adult populations in countries such as the United States, Canada and New Zealand^(43)^, surpassing rates observed in the non-Indigenous populations in these countries. Transformations in diets, loss of territories and accelerated urbanisation are among the factors identified as connected to the accelerated nutrition transition observed in Indigenous populations in diverse world regions^(44,45)^.

Until recently, the nutritional and health situation of Indigenous peoples in Brazil was known only through specific village or community case studies due to the absence of nationwide surveys. Consequently, it was difficult to generalise about nutrition transition among the country’s ethnically diverse Indigenous peoples^(34)^. The present National Survey, which is the largest health study of the Indigenous population in Brazil yet conducted based on a representative sample at national and regional levels, shows that the levels of excess weight in Indigenous women are similar to the national nutritional transition profiles^(46)^. Whereas 46·2 and 15·8 % of Indigenous women evaluated in the present study presented excess weight and obesity, respectively, these values were 48·0 and 16.9 % for all adult Brazilian women based on a national study conducted in 2008–9^(46)^. Comparing the results of nationally representative surveys since the 1970s, the prevalence of obesity among adult women generally has increased by 2·3 times over approximately four decades (7·2 % in 1974–5 and 16·9 % in 2008–9)^(9,46)^. There are no nationally representative data on the progression of excess weight and obesity through time for Brazil’s Indigenous population. However, it is known from case studies conducted in specific communities that the onset and evolution of obesity is a relatively recent and accelerated process. This conclusion is deduced from the relative absence of studies highlighting excess weight and obesity in the health profile of Indigenous populations until the 1990s^(24,28–30)^.

The National Survey also permits the first comparisons between Brazil’s major geopolitical regions (North, Central-West, Northeast and South/Southeast). Stratification of the occurrence of excess weight and obesity according to these four regions reveals a scenario of extremes of great epidemiological and socio-anthropological significance. Whereas obesity affected 22·6 % of women in the South/Southeast, the prevalence observed in the North was almost four times less, at 6·1 %. Also, the South/Southeast and Central-West regions presented the highest frequencies of excess weight, with more than half of Indigenous women affected (54·7 and 52·8 %, respectively). In contrast, the prevalence of excess weight in the North was 31·2 %. These comparisons highlight important interregional differences in the nutritional profiles of Indigenous women in Brazil, such as between the North, dominated by Amazonia, and the South/Southeast, which includes the country’s largest metropolitan regions. They also show that even in the North, where the lowest levels of excess weight and obesity were encountered, there is strong evidence of nutrition transition, as previously documented by local case studies^(24,29)^.

Similar geographical distributions among the Indigenous populations in Brazil have been described for other chronic diseases such as arterial hypertension and diabetes mellitus. In a previous paper presenting the findings of the National Survey, 13·2 % of Indigenous women nationwide were found to present hypertension, while this prevalence was just 3·6 % in the North^(34)^. Compared to the North, hypertension prevalence was about three times higher in the Northeast and five times higher in the South/Southeast and Central-West. Similarly, the nationwide prevalence of high blood glucose values, suggestive of diabetes mellitus, in women was 1·4 %, ranging from 0·5 % in the North to 2·1 % in the South/Southeast^(34)^. Although the observed patterns of geographical contrasts are similar for hypertension, high blood glucose, excess weight and obesity, the scales are quite different. With excess weight and obesity showing much higher frequencies than hypertension and high blood glucose, it may be concluded that the nutritional pattern observed in Indigenous women nationwide reflects the nutrition transition trajectory documented in other Indigenous and non-Indigenous populations, whereby a widespread occurrence of obesity precedes the entrance of hypertension and diabetes.

Explanations of the expressively lower levels of excess weight and obesity in the North compared to other regions may involve relatively direct ecological and behavioural determinant factors, especially those related to food environments and physical activity. For example, the North stands out from all other regions for having much higher numbers of villages that reported acquiring food by hunting, fishing and collecting^(34)^. Less directly, the interregional contrasts documented by the National Survey are historically derived from the geography of Brazil’s expanding demographic and economic frontiers. Exemplifying this process and its implications for contemporary Indigenous peoples is the distribution of federally recognised Indigenous lands, which occupy about 13 % of the Brazilian territory. Of the country’s 117 067 410 ha of Indigenous reserves, 98 % by area is in the Amazon, which predominates in the North and the northern portion of the Central-West region. The remaining 2 % are scattered throughout other regions in the country, with the smallest and most discontinuous lands concentrated in the southern and coastal regions that were the first to be colonised by European settlers. The Indigenous peoples living in the North often retained greater access to territories and traditional landscape resources as a result of more recent histories of contact and social integration, along with late-twentieth-century public policies that were more favourable to the recognition of Indigenous land rights and the preservation of larger Indigenous reserves. The National Survey finding that food acquisition by hunting, fishing and collecting is most prevalent in the North is likely related to the historical contrasts in the country’s distribution of indigenous lands^(34)^. Consistent with these previously published findings and explanations, bivariate analyses in the present study found the highest frequencies of excess weight and obesity in the Northeast and South/Southeast regions and in households that did not report consuming foods obtained by hunting, fishing and collecting.

In the growing academic production of explanatory models for nutrition transition in Indigenous populations in different parts of the world, dietary and lifestyle changes (e.g. physical inactivity) have been highlighted as important factors associated with increased levels of excess weight and obesity in adults^(13,44,47)^. Due to the diversity of local and regional sociohistorical contexts in different parts of the world, caution is warranted in attempting to advance general explanatory models for the environmental, political and economic factors associated with the nutrition transition observed in many Indigenous populations. Nevertheless, an emerging pattern among many Indigenous peoples, supported by ample case studies in diverse world regions, is that greater integration in market economies tends to be associated with increased prevalence of excess weight and chronic non-communicable diseases such as diabetes and hypertension^(48,49)^. The results of the multivariate statistical modelling presented here show associations that align closely with these explanatory models. In addition to excess weight and obesity differing according to geopolitical region, as discussed above, the results of multivariate models presented here also show that Indigenous women living in households that report purchasing foods are four times more likely to be obese. There was also an increased chance of obesity in women living in households with higher household goods index scores and higher housing conditions index (reflecting greater market insertion in terms of construction materials, access to electricity and cooking fuel).

The present results of the National Survey point to distinct patterns of associations between socioeconomic indicators and the occurrence of excess weight and obesity among Indigenous women. For example, whereas the household goods index and the existence of regular household income were positively associated in bivariate analyses with excess weight and obesity, more years of schooling was a protective factor for both outcomes. A similar scenario involving inverse relationships between income and schooling and the outcomes excess weight and obesity has also been described for the general adult population^(50)^. In a comparative study on nutritional transition patterns in two regions of Brazil, Monteiro *et al*.^(50)^ observed that ‘income tends to be a risk factor for obesity while education tends to be protective and that both gender and level of economic development are relevant modifiers of the influence exerted by these variables’ (p. 885s). However, it is noteworthy that this difference was less accentuated in the multivariate models in the present study, which did not retain regular income as a significant variable, and in which only ≥10 years of schooling acted as a protective factor.

Several notable independent variables were significantly associated with excess weight and/or obesity in bivariate analyses but lost significance in the multivariate multilevel analyses. At the village level, food acquisition activities (hunting, fishing and collecting) and presence of a school meals programme were not retained in either final model. At the household level, one of the two household food acquisition activities (horticulture/livestock) was not retained, nor the composite purchased foods index derived through the principal component analyses. Similarly, access to the staple foods donation programme and use of oils and fats for cooking lost significance in both models. The only household food access indicator retained in a final model was food acquisition by purchasing, an indicator serving to distinguish households that report not buying foods from those that report buying them. This pattern suggests that village-level food economic indicators were less sensitive for excess weight and obesity than household-level indicators. It also shows that indicators more closely related to market insertion (household goods index and purchasing food) were more directly determinant of obesity than those emphasising food economies and subsistence activities.

Although the National Survey represents an important advance in the study of Indigenous peoples’ health in Brazil because it was representative at the national and regional levels, it should be noted that it was not designed to capture contrasts in nutritional profiles between specific Indigenous ethnic groups. As documented in the most recent Demographic Census conducted in Brazil in 2010, there are approximately 300 Indigenous ethnic groups in the country^(51)^, making Brazil one of the most ethnically diverse countries in the Americas and the world. This scale of national socio-diversity suggests the possibility of innumerable idiosyncratic profiles of engagement with Brazilian non-Indigenous society, each of which may involve distinct environmental or socioeconomic factors with the potential to influence nutritional transition, generally, and excess weight and obesity, specifically. In this sense, the findings presented here do not directly represent any singular Indigenous ethnic group in Brazil. Rather, the study was intended to capture broader frames of reference, the results of which suggest possible avenues of research for future medium (regional or ethnic group) to small-scale (community) surveys^(34)^. Nevertheless, the community studies conducted over the past two decades in different ethnic groups throughout the country’s major geopolitical regions point to excess weight and obesity as generally prominent issues in the nutritional profile of adult Indigenous women. For example, women from numerous specific communities and ethnic groups presented prevalence of obesity ≥20 %, such as the Xavante, Terena, Guarani and Kaiowá in the Central-West^(26,30,52)^, the Xukuru in the Northeast^(28)^, the Suruí in the North^(29)^ and the Kaingang and Guaraní-Mbyá in the South/Southwest^(53,54)^.

Several other potential limitations of the study should be noted. According to its original formulation, the National Survey focused on Indigenous women and children, not adult men, due to its primary emphasis on child and maternal health. The study therefore also excluded adolescents and elders of both sexes. Additionally, the list of villages used to select the sample population included only those located within federally recognised Indigenous reserves served by the Indigenous Healthcare Subsystem. Also, considering that 5714 (after exclusion) of 6692 participating women were analysed, the possibility of selection bias may be negligible. Furthermore, because data were collected from 2008 to 2009, they might not fully capture the present-day scenario. Finally, the cross-sectional nature of the study precludes assessing diachronic nutrition transition patterns or infer causality from associated risk factors.

In conclusion, this study indicates that higher socioeconomic indices, market-integrated living conditions and less reliance on local food production, as well as increased age and parity are associated with excess weight and obesity in adult indigenous women in Brazil. These findings from the National Survey have potentially significant implications from a public policy perspective for Indigenous peoples in Brazil. Frequencies of excess weight and obesity were found to be lower in regions (such as the North) where Indigenous populations have greater access to traditional territories. Access to land is an important condition for the continuation of traditional subsistence practices, potentially impacting not only dietary patterns but also physical activity. Another central issue is the need to consider the results in the context of public policies implemented by the Brazilian government aimed at improving food and nutrition security. Many of these policies have been based on governmental cash transfer programmes. For the general non-Indigenous Brazilian population, there is ample evidence that such initiatives are linked to the improvement of children’s living conditions, as well as greater food security of beneficiary families^(55)^. However, in the case of Indigenous peoples, the few studies that have addressed these initiatives have not focused on their potential impacts on child or adult nutritional profiles^(56)^.

Finally, it is important to emphasise the need to carry out studies to monitor changes in the nutritional situation of the Indigenous population through time, particularly considering the rapid nutritional transition in progress. In Brazil, the challenge remains to produce reliable nationally representative statistics at regular intervals on the health conditions of ethnic minorities, including Indigenous peoples^(57)^, who often live in environmentally, nutritionally and socioeconomically vulnerable conditions.
